# Cation‐Tuning Engineering on Metal Oxides for Oxygen Electrocatalysis

**DOI:** 10.1002/chem.202202000

**Published:** 2022-11-28

**Authors:** Liuyue Cao, Bin‐Wei Zhang, Shenlong Zhao

**Affiliations:** ^1^ School of Chemistry and Chemical Engineering Chongqing University Chongqing 400044 P. R. China; ^2^ Center of Advanced Energy Technology and Electrochemistry Institute of Advanced Interdisciplinary Studies Chongqing University Chongqing 400044 P. R. China; ^3^ School of Chemical and Biomolecular Engineering The University of Sydney Sydney New South Wales Australia

**Keywords:** cation tuning, electronic structure, oxygen evolution reaction, oxygen reduction reaction, Zn air batteries

## Abstract

Cation‐tuning engineering has become a new frontier in altering the electronic structure of electrocatalysts, which has been employed to enhance their electrochemical performance. Significant efforts have been made to promote the electrochemical performance of transition metal‐based materials during oxygen electrocatalysis and related energy devices such as Zn‐air batteries. Herein, the advantages of cation‐tuning engineering, including cation vacancies/defects and cation doping, in the modification of the electronic structure of transition metal oxide catalysts are discussed. Additionally, practical applications of the cation‐tuning engineering strategy are reviewed in detail with a special emphasis on oxygen reduction reaction and oxygen evolution reaction. Lastly, challenges and future opportunities in this field are also proposed.

## Graphical abstract

**The cation‐tuning engineering** strategy as a new emerging frontier has attracted significant attention for transition metal‐based catalysts. Its advantages are summarized with special emphasis on altering the electronic structure and charge distribution of transition metal‐based catalysts. This Concept also reviews its application for the oxygen reduction reaction, the oxygen evolution reaction, and Zn air batteries, and also proposes future opportunities.

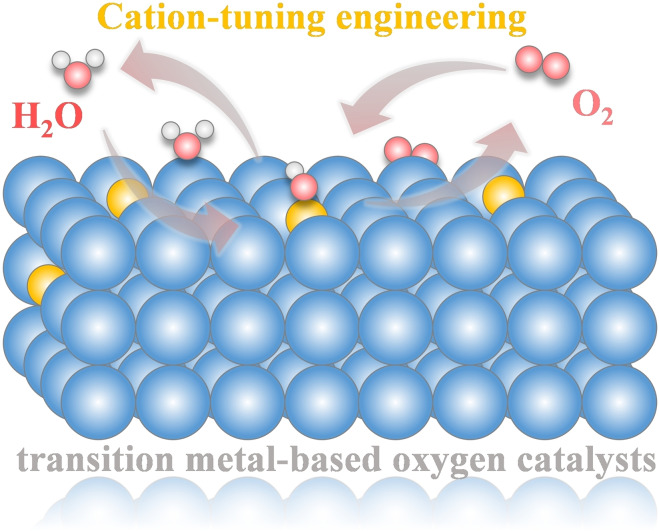

## Introduction

Nowadays, renewable energy technologies, such as fuel cells, metal‐air batteries, and water splitting, are considered promising strategies to solve the current energy challenges.^[1]^ These energy‐related systems are usually involved in oxygen reduction reaction (ORR) and oxygen evolution reaction (OER).^[2]^ Specifically, ORR is the cathodic reaction for fuel cells and metal‐air batteries;^[3]^ OER is the inverse reaction of ORR, which is an important process of water splitting and metal‐air batteries.^[4]^ Nevertheless, both reactions suffer from sluggish kinetic due to their multi‐step electron transfer process and thus inhibit their practical applications. To enhance the kinetic of oxygen reactions, great efforts have been devoted to improving the activity of catalysts and revealing their intrinsic structure‐activity relationship.^[5]^ In comparison to noble metal‐based catalysts, including Pt, RuO_*x*_, and IrO_*x*_, transition metal‐based materials are promising catalysts for ORR and OER because of their low cost and abundance of resources.^[6]^ However, the electrochemical performance of these transition metal‐based catalysis is still not comparable with those of noble metal‐based catalysts due to a lack of rational design.^[7]^

Recently, an efficient strategy was developed to enhance their kinetic on transition metal‐based catalysis via tuning cation vacancies/defects and cation doping, which is expected to modulate the electronic structure of metal oxide and thus achieve high performance towards ORR and OER.^[8]^ This cation vacancies/defects engineering can regulate the d‐band center of catalysts, which affects the adsorbed/desorbed configuration and corresponding energy of intermediates, thereby influencing their ORR and OER performance.^[9]^

Herein, the advantages of cation‐tuning engineering, including cation vacancies/defects and cation doping, of transition metal oxide catalysts in modifying their electrochemical performance were discussed. Then, the applications of cation‐tuning engineering are summarized in detail with a special emphasis on ORR, OER, and oxygen‐involved batteries. Finally, current challenges and future opportunities for the development of cation vacancies/defects engineering for metal oxides are highlighted.

## The Advantages of Cation‐tuning Engineering

Recently, cation‐tuning engineering has been developed as an efficient strategy to tune the electronic structure of transition metal oxide catalysts for electrochemical performance enhancement. It is well known that the state‐of‐the‐art of ORR and OER electrocatalysts are noble metal‐based catalysts.^[10]^ However, the high price and limited reserve in nature inhibit their large‐scale industrial application.^[11]^ Transition metal‐based materials have attracted significant attention due to their low cost and promising electrochemical performance.^[12]^ To date, great efforts have been made to improve the 3d transition metal‐based catalysts, such as size regulation (e. g. single‐atom catalysts),^[13]^ heterometal atoms doping,^[14]^ defect engineering,^[15]^ etc. It should be noted that the activity of the transition metal‐based catalysts has a close relationship with the position of their d‐band center (*E*_d_);^[16]^ that is, the adsorption and desorption of intermediates can be tuned by modifying the *E*_d_ of catalysts. Therefore, it is urgently required to develop effective methods to tune the electronic structure of 3d transition metal‐based catalysts to improve their electrochemical activity.

**Figure 1:**
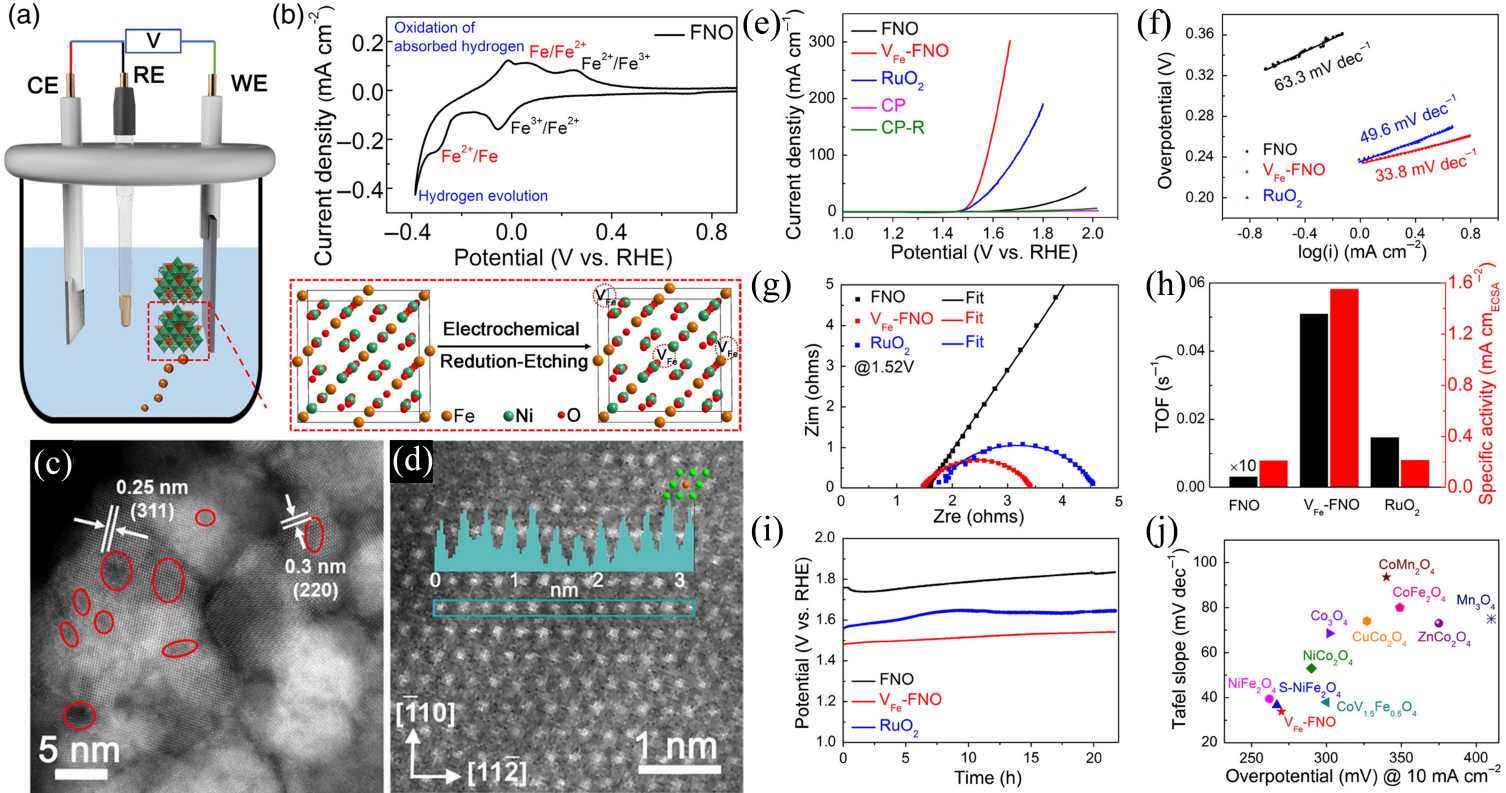
(a) ‐ (b) Schematic illustration for the synthesis of the Fe cationic defects of spinel‐type FeNi_2_O_4_ (V_Fe_‐FNO). (c)–(d) Scanning transmission electron microscopy (STEM) imaging of V_Fe_‐FNO. (e)–(j) Electrochemical performance of V_Fe_‐FNO for OER in 1.0 M KOH. Reproduced with permission.^[8]^ Copyright 2020, Chinese Chemical Society.

Cationic tuning engineering can be achieved by atomic scale regulation to modify the electronic structure of metal oxide catalysts for ORR/OER applications. Chen et al. reported an electrochemical reduction etching method to tune the cationic engineering of spinel‐type FeNi_2_O_4_ (FNO) (Figure 1).^[8]^ Scanning transmission electron microscopy (STEM) imaging in Figures 1c and 1d demonstrates that the obtained defective FeNi_2_O_4_ (V_Fe_‐FNO) has many cationic defects. Interestingly, these Fe cationic defects can not only tune the electronic structure of FNO to modify the adsorption energy of intermediates, but also enrich active sites; therefore, the FNO presented a low overpotential of 270 mV and a Tafel slope of 33.8 mV dec^−1^ at 10 mA cm^−2^ towards OER in 1.0 M KOH, outperforming the commercial RuO_2_ (Figure 1e–1j).

**Figure 2:**
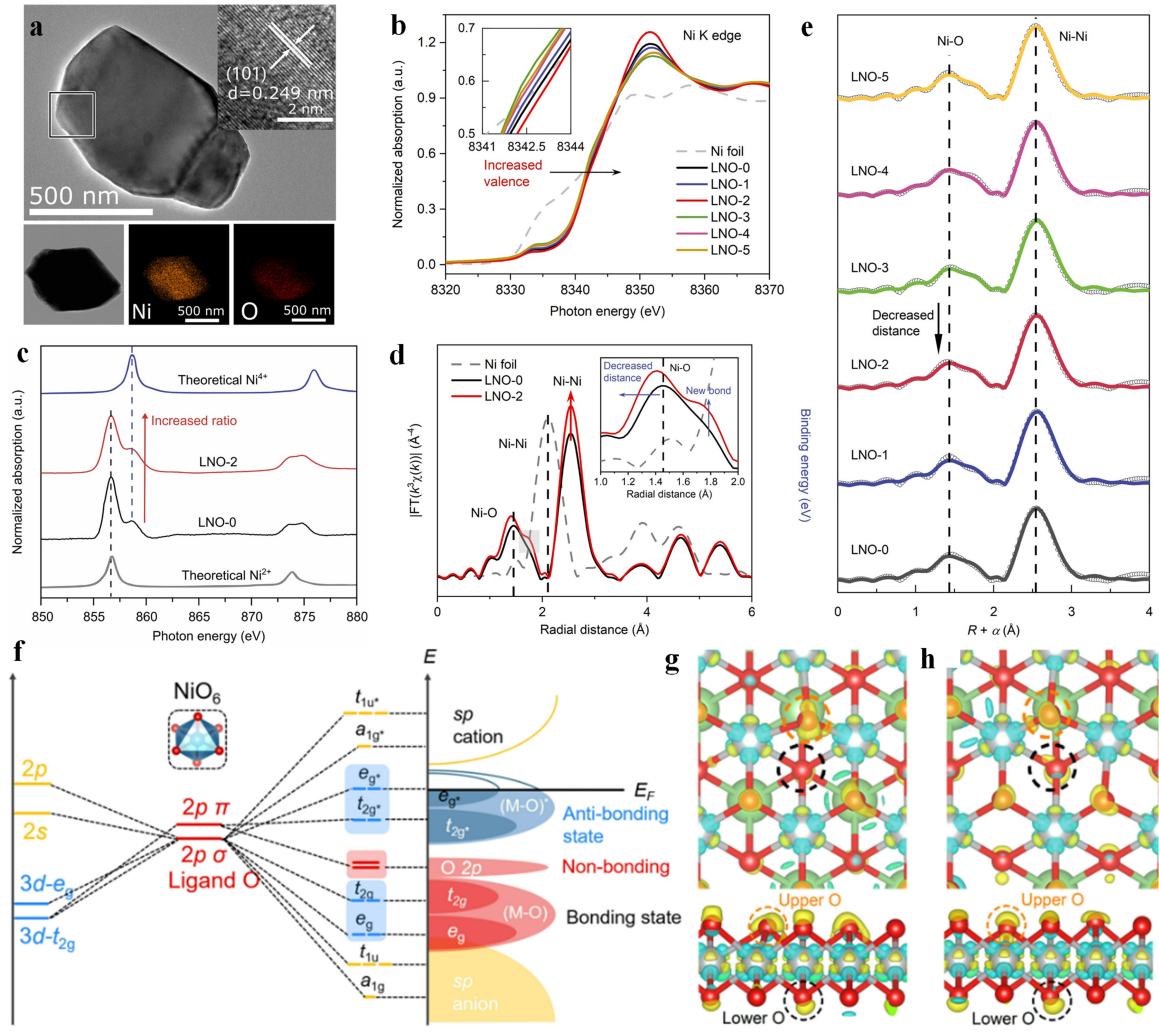
(a)TEM images with elemental mapping of LNO. (b) Normalized Ni K‐edge X‐ray Absorption Near Edge (XANE) Structure spectra of LNO‐x. (c) Ni *L*‐edge soft X‐ray absorption spectroscopy (sXAS) spectra and (d) Extended X‐ray absorption fine‐structure (EXAFS) *k* ^3^ *χ*(*R*) Fourier transform (FT) spectra of LNO‐0 and LNO‐2. (e) Ni K‐edge EXAFS k3χ(R) spectra (points) and fitting results (line) of LiNO2‐x. (f) Molecular orbital energy diagram for octahedral NiO_6_. Differential charge densities of LNO‐0 (g) and LNO2 (h). Reproduced with permission.^[17]^ Copyright 2022, Wiley‐VCH.

Cationic tuning engineering can also redistribute the charge of metal, resulting in the shift of charge‐transfer energy, and thus enhancing the electrochemical activity.^[9]^ For example, Han et al. prepared various delithiated LiNiO_2_ (LNO‐*x*, *x*=0‐5) catalysts through a chemical delithiation strategy.^[17]^ The delithiated process created charge unbalances and induced the rearrangement of the edge oxygen electron density (Figure 2), resulting in a new non‐bonding O 2*p* band near the M−O, pushing Ni^2+^ and Ni^3+^ from the Fermi level, which facilitated the deprotonation in lattice oxygens during catalytic reactions.

## The Electrochemical Application of Cation‐Tuning Engineering

### ORR

**Figure 3:**
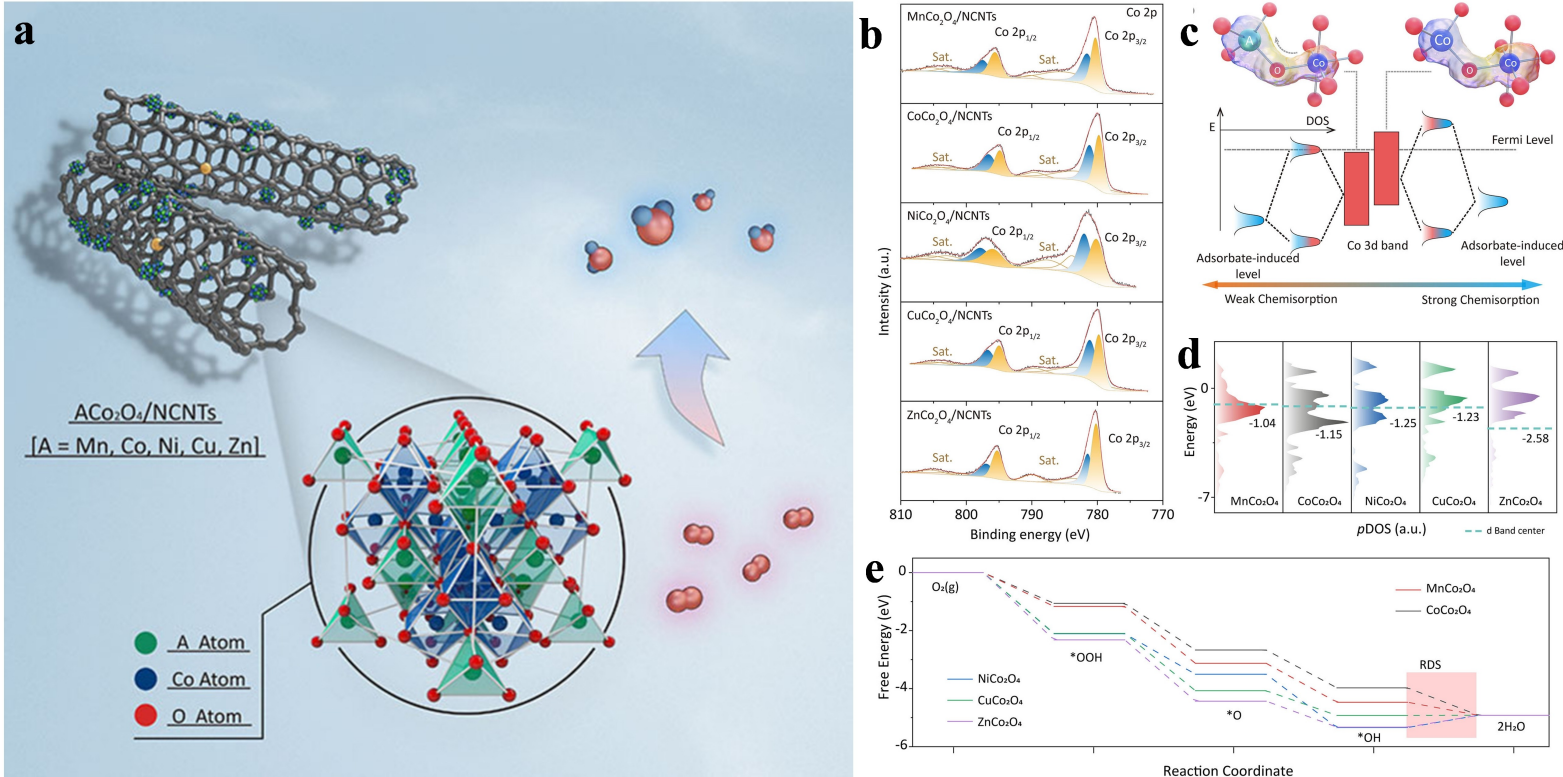
(a) Schematic illustration of ACo_2_O_4_/NCNTs (A=Mn, Co, Ni, Cu, Zn) for ORR. (b) XPS spectra of Co 2p in ACo_2_ centers. (c) Schematic diagram of tetrahedral and octahedral interaction, (d) d‐band center, and (e) the calculated free‐energy diagram of ACo_2_O_4_/NCNTs. Reproduced with permission.^[24]^ Copyright 2022, Wiley‐VCH.

Fuel cells are green and efficient energy technologies that convert chemical energy to electricity.^[18]^ However, the slow kinetics of cathodic reaction ORR is the main challenge to its commercialization.[^5^, ^19^] It generally has two reaction pathways: a two‐electron reduction to H_2_O_2_ and a four‐electron pathway to H_2_O.^[20]^ The former mechanism is desired for the electrosynthesis of chemical hydrogen peroxide while the latter one is favored for the fuel cell and even metal‐air batteries. A typical spinel oxide (AB_2_O_4_) consists of an octahedrally coordinated B point (M_Oh_) and a tetrahedrally coordinated A point (M_Td_), which can share the same or different transition metals.^[21]^ Particularly, M_Oh_ in the spinel framework plays an important role in the electrochemical performance of oxygen‐related reactions.^[22]^ Thus, altering the M_Oh_ by introducing cation‐tuning strategy is expected to improve the ORR performance of AB_2_O_4_.^[23]^ Wang et al. employed a simple solvent method to optimize the charge structures of ACo_2_O_4_ (A=Mn, Ni, Co, Zn, Cu) via modifying the Co_Oh_ (Figure 3).^[24]^ For a typical CoCo_2_O_4_, the O serves as an “electron bridge” to generate an efficient Co_Td_−O−Co_Oh_ electron transport pathway in Co‐based spinel, and Co^3+^_Oh_ can act as the active center and the oxygenated species for ORR and OER. By introducing hetero‐elements, the charge structure of Co^3+^_Oh_ was tuned via modifying their d‐band centers, thus affecting the ratio of Co^3+^/Co^2+^. The electron transfer process of these ACo_2_O_4_ was elaborated by X‐ray photoelectron spectroscopy (XPS) and DFT (density functional theory) and the Mn element was shown to have the best adjustment effect. Therefore, an outstanding Zn‐air battery was enabled by MnCo_2_O_4_ with a high‐power density of 74.63 mW cm^−2^ and no obvious degradation was observed over 300 cycles at the 5 mA cm^−2^.

**Figure 4:**
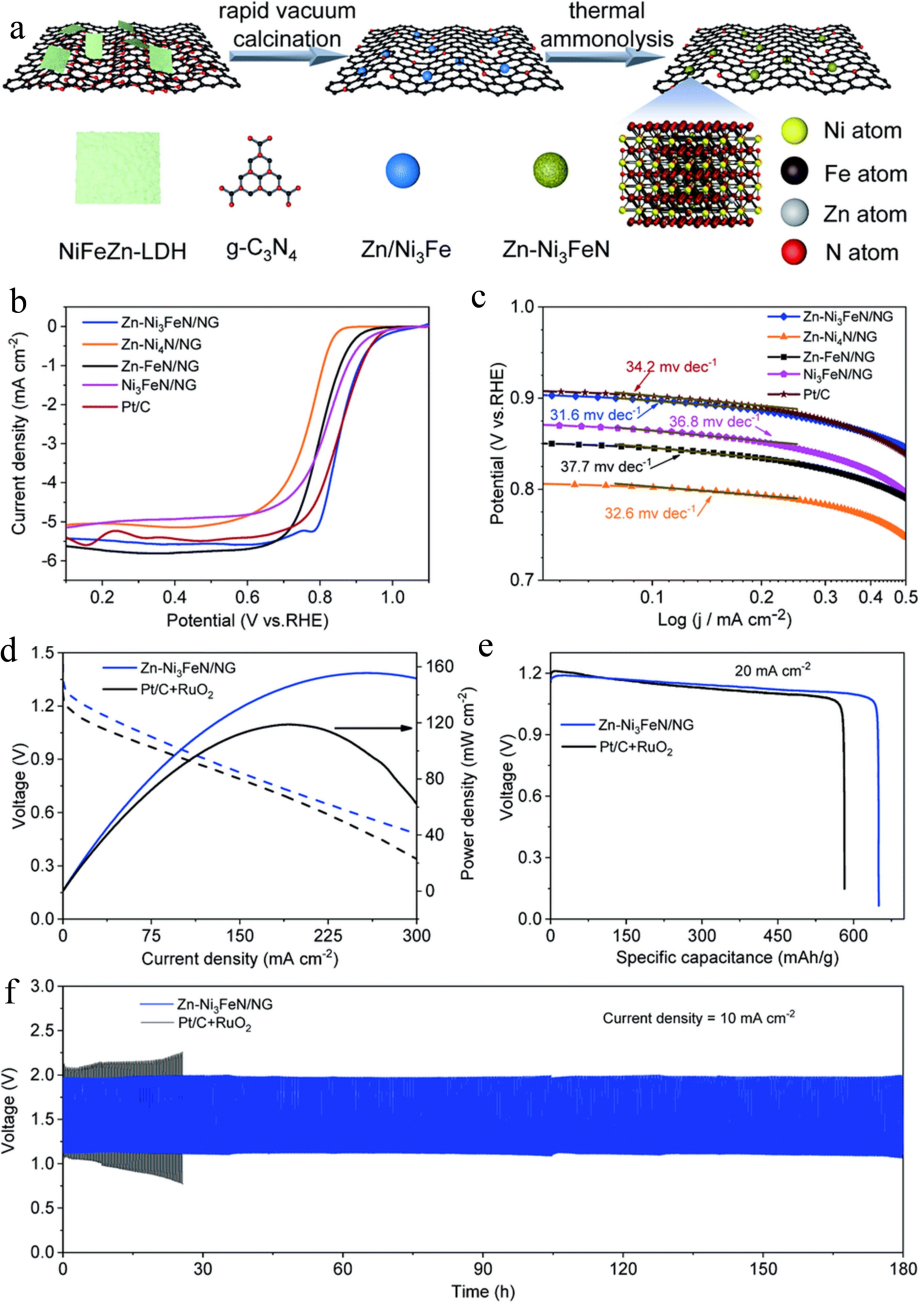
(a) Schematic illustration of the synthesis of Zn‐Ni_3_FeN/NG; (b) ORR performance and (c) Tafel plots of Zn‐Ni_3_FeN/NG, Zn‐Ni_4_N/NG, Zn‐FeN/NG, Ni_3_FeN/NG and Pt/C, 1600 rpm, at 10 mV s^−1^ in O_2_‐saturated 0.1 M KOH; (d) charge and discharge polarization curves, (e) discharge curves at 20 mA cm^−2^, and (f) long‐term cycling performance at 10 mA cm^−2^ of Zn‐Ni_3_FeN/NG and Pt/C+RuO_2_ as air cathodes for Zn‐air batteries. Reproduced with permission.^[26]^ Copyright 2021, The Royal Society of Chemistry.

Besides, cation tuning engineering can be used to transition metal‐based to alter their electronic environment and create more active sites. Bimetallic nitrides usually show an outstanding performance for OER due to their two active sites, and electronic states.^[25]^ Nevertheless, the poor ORR performance of bimetallic nitrides inhibits the application of Zn‐air batteries. He et al. introduced a third metal into bimetallic nitrides as the heteroatom to boost their ORR catalytic activity.^[25]^ They designed Zn‐doped Ni_3_FeN/nitrogen‐doped graphene (Zn‐Ni_3_FeN/NG) as electrocatalysts for rechargeable Zn‐air batteries (Figure 4).^[26]^ The inserted Zn^2+^ cation ions increased the surface electronic states of Ni_3_FeN/NG to near the Fermi level, altered the electronic structure of Ni_3_FeN and modulated the adsorption/desorption behavior, thereby reducing the reaction barriers for ORR. Therefore, a remarkable bifunctional performance for ORR and OER was achieved and the Zn‐air battery with this catalyst exhibited a high‐power density of 158 mW cm^−2^.

Cationic engineering also can be applied to alter the electronic structure of metal phosphide. For example, Zhou et al. reported Ni cationic vacancies (V_Ni_)‐enriched Ni_2−*x*_P‐V_Ni_ electrocatalyst for H_2_O_2_ generation via ORR.^[27]^ Compared with V_Ni_‐free Ni_2_P (without V_Ni_), the density of state (DOS) of Ni_2−*x*_P‐V_Ni_ electrocatalyst was near the Fermi level at Ni 3d, rather than P 3p; in addition, the *E*_d_ of Ni_2−*x*_P‐V_Ni_ electrocatalyst was at −2.48 eV, positive‐shifted compared with that of V_Ni_‐free Ni_2_P (−2.53 eV). The modified electronic structure of the Ni_2−*x*_P‐V_Ni_ electrocatalyst optimized the intermediate *OOH, and thus facilitated the formation of H_2_O_2_.

### OER

**Figure 5:**
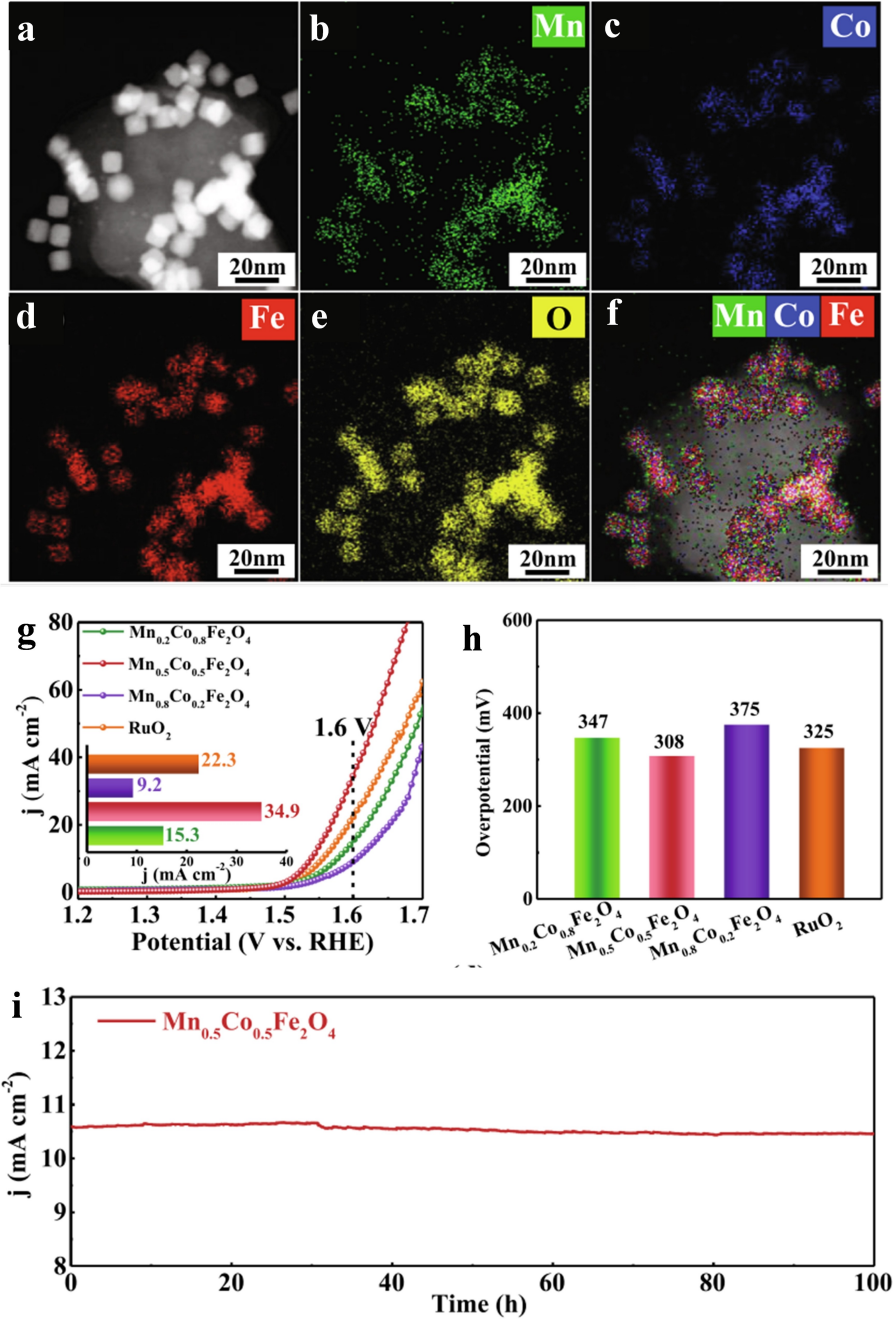
(a)–(f) HAADF‐STEM images and elemental mapping of Mn_0.5_Co_0.5_Fe_2_O_4_. (g)‐(h) The OER performance of trimetallic cubic Mn_x_Co_1−x_Fe_2_O_4_ and commercial RuO_2_ catalysts in O_2_‐saturated 1.0 M KOH. (i) Durability test of Mn_0.5_Co_0.5_Fe_2_O_4_ within 100 h at 1.54 V in O_2_‐saturated 1.0 M KOH. Reproduced with permission.^[29]^ Copyright 2022, Elsevier Ltd.

OER is a process of generating O_2_ from water oxidation, which has been widely studied because it is the rate‐limiting step in various electrochemical energy devices, (e. g., water electrolysis systems).^[28]^ As the OER process involves the adsorption/desorption of oxygen intermediates, cation‐tuning engineering can be an excellent approach to enhance their kinetics. To modify the d‐band center of transition metal‐based spinel oxide materials, ternary or even quaternary spinel oxides can be employed to boost the OER performance. For example, Zeng et al. used a synthetic method to dope Co and Mn into inverse spinel Fe oxides to form ternary cubic inverse spinel Mn_0.5_Co_0.5_Fe_2_O_4_,^[29]^ as shown in the scanning transmission electron microscope‐X‐ray energy dispersive spectroscopy (STEM‐EDS) element mapping images (Figure 5). Such doping can optimize the bond strength with key intermediate (OOH*) for OER, and thus decrease its energy barrier. Therefore, it exhibited a high current density of 34.9 mA cm^−2^ at 1.6 V even higher than that of commercial RuO_2_ (22.3 mA cm^−2^). In addition, this ternary Mn_0.5_Co_0.5_Fe_2_O_4_ catalysts also showed excellent stability with only 2.84 % decrease at 1.54 V for 20 h.

**Figure 6:**
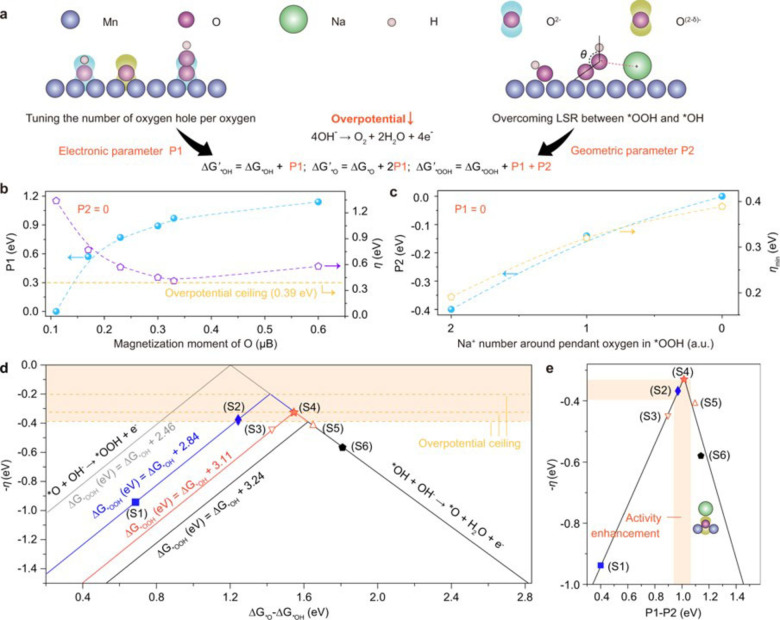
(a) Schematic illustration of the design of Na_*x*_Mn_3_O_7_ electrocatalysts. (b) The shift of electronic parameter (P1) to modify the theoretical overpotential (η). (c) The shift of geometric parameter (P2) to decrease the overpotential ceiling (η_min_). (d) Dynamic volcano plot for ORR mechanism. (e) Unified volcano plot for Na_*x*_Mn_3_O_7_ electrocatalysts. Reproduced with permission.^[31]^ Copyright 2021, Nature Publishing Group.

Apart from transition metal, alkali metal ion (A^+^) also can be used to AB_2_O_4_ to stabilize the key intermediate productions through the noncovalent interaction.^[30]^ Huang et al. employed Na_*x*_Mn_3_O_7_ materials as modeled catalysts to investigate its OER performance.^[31]^ They found that Na^+^ formed a chemical bond with oxygen, which can tune the oxygen lone‐pair states and then modify the energy barriers of O−H bond breakage and *OOH key intermediates formation (Figure 6). The regulation of the OER activity of Na_*x*_Mn_3_O_7_ by these cation vacancies was also supported by their in situ Raman and DFT results.

**Figure 7:**
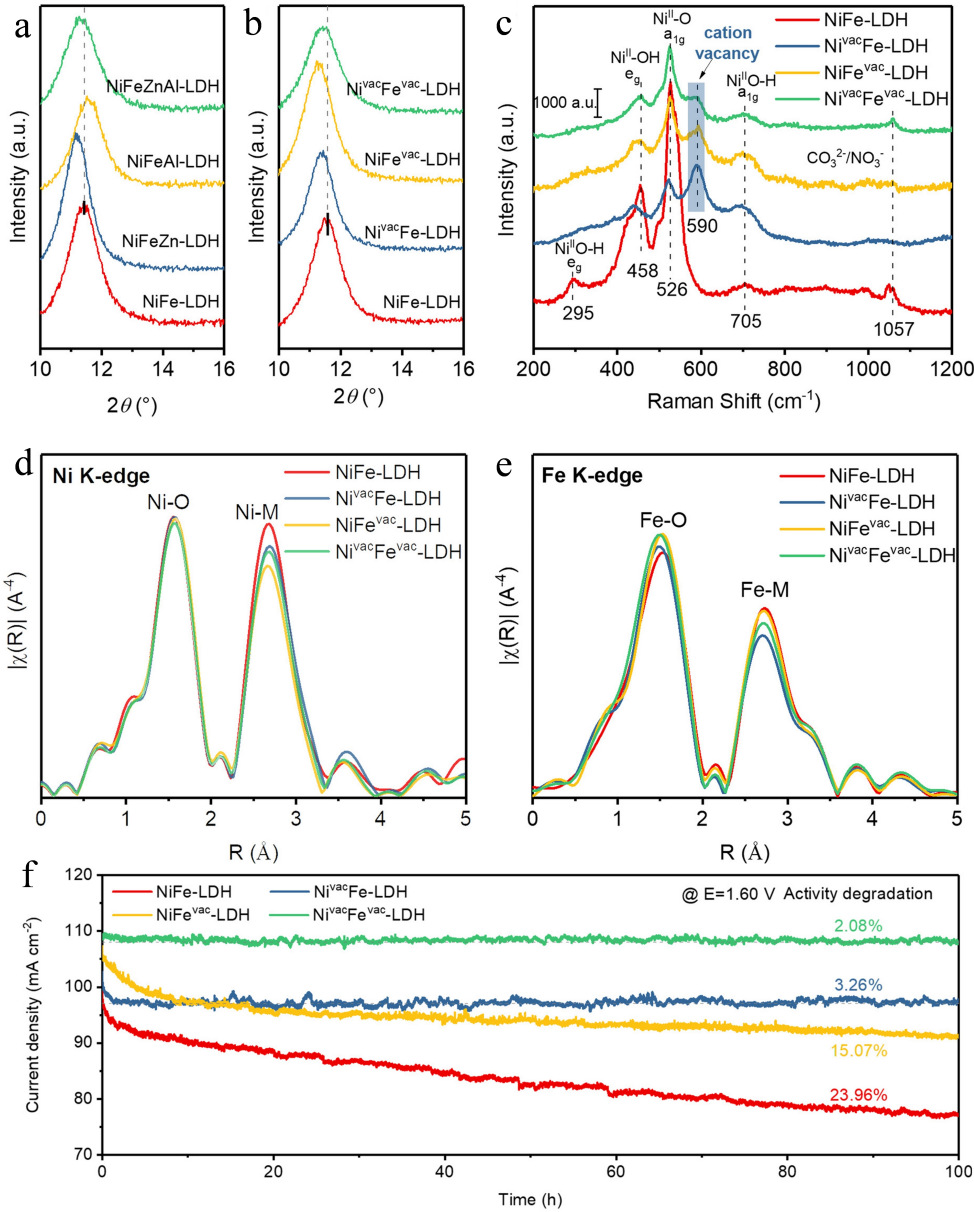
(a)–(b) X‐ray diffraction (XRD), (c) Raman spectra (d)–(e) Ni K‐edge and Fe K‐edge of NiFe‐LDH‐based materials. (f) OER performances of NiFe‐LDH‐based materials catalysts in O_2_‐saturated 1.0 M KOH. Reproduced with permission.^[36]^ Copyright 2021, Wiley‐VCH.

NiFe oxide‐based materials are promising OER catalysts, and have been widely studied. For example, nickel‐iron layered double hydroxides (NiFe‐LDHs) are promising catalysts for OER in an alkaline medium.^[32]^ Although a large number of strategies, including defect engineering,^[33]^ metal doping,^[34]^ etc, have been utilized to modify their surface structure and composition, the stability of NiFe‐LDHs still fails to meet the requirements of practical application.^[35]^ Metal dissolution is the major reason for the activity degradation of NiFe‐LDHs; therefore, it is expected that introducing cation vacancies will enhance the binding energy between Ni and Fe, thus increasing the stability of NiFe‐LDHs. For example, Peng et al. claimed that cation vacancies can accelerate the evolution of surface γ‐(NiFe)OOH active species, maintaining the stability of NiFe‐LDHs^[36]^ (Figure 7). They found that the cation (M^2+^) vacancies strengthened the connection between adjacent metal cations and oxygen, while the cation (M^3+^) vacancies accelerated the formation of γ‐(NiFe)OOH phases, improving both their activity and stability. Li reported that doping vanadium cationic ion into NiFe LDHs will tune its electronic structure, and shorten the bandgap of NiFe LDHs, thus increasing its conductivity and promoting electron transfer.^[37]^

**Figure 8:**
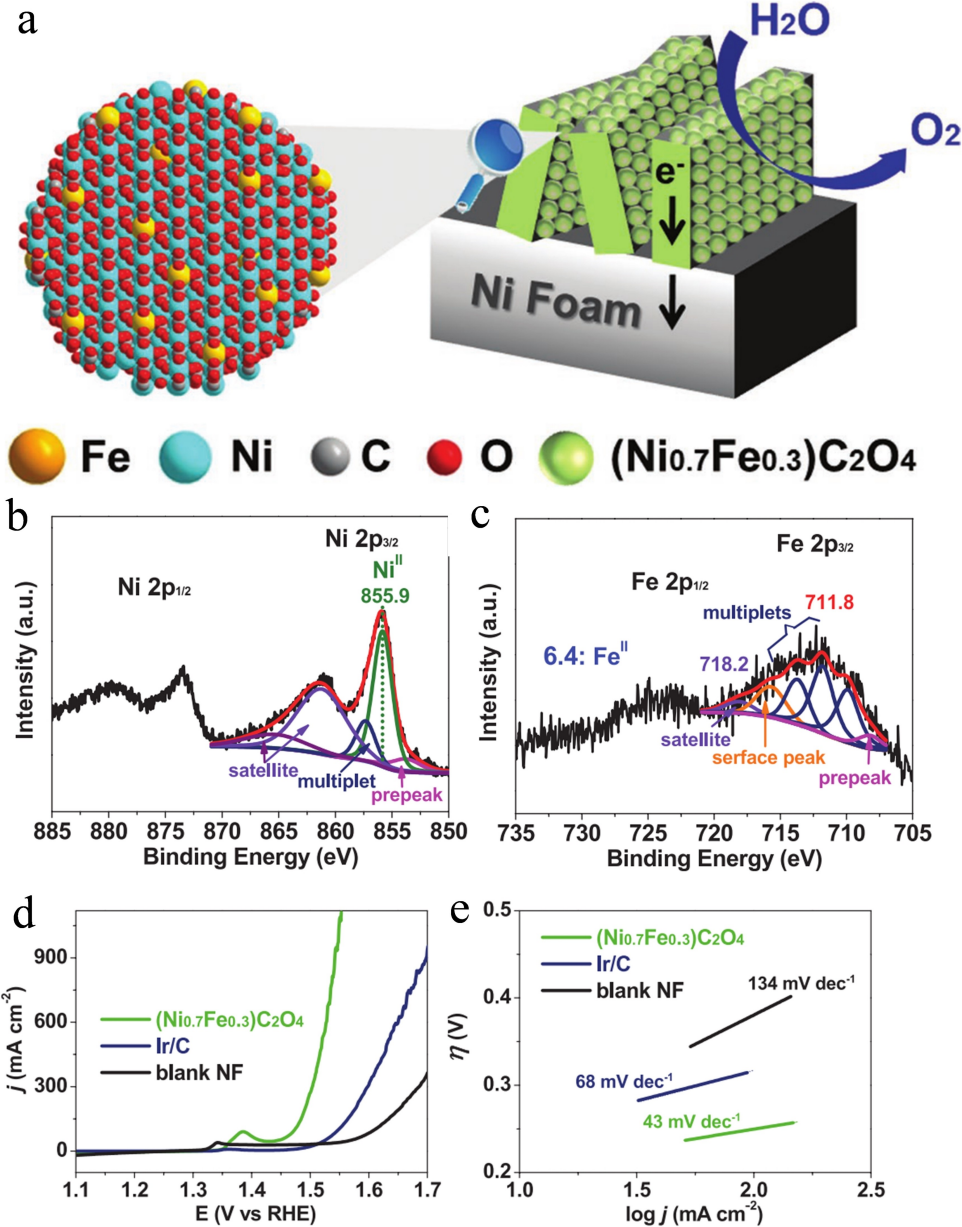
(a) The illustration of (Ni_0.7_Fe_0.3_)C_2_O_4_ catalyst for OER. (b) Ni 2p and (c) Fe 2p XPS spectra of the (Ni_0.7_Fe_0.3_)C_2_O_4_ catalyst. (d) LSV polarization curves and (e) Tafel plots in O_2_‐saturated 1.0 M KOH of (Ni_0.7_Fe_0.3_)C_2_O_4_, nickel foam (blank NF), and Ir/C. Reproduced with permission.^[37]^ Copyright 2019, Wiley‐VCH.

NiFe oxalate also has been employed as OER catalyst.^[38]^ However, the typical NiFe oxalate usually suffers from phase separation, and thus reducing the electrochemical activity towards OER.^[39]^ Gao et al. reported oxalate anions to capture Fe^3+^ into Fe^2+^, and form a homogeneous Fe cationic doping of (Ni_0.7_Fe_0.3_)C_2_O_4_ catalyst, which showed great potential application towards OER, as shown in Figure 8.[^37^, ^40^] XPS of (Ni_0.7_Fe_0.3_)C_2_O_4_ catalyst indicated the successful preparation of Fe cationic doping. It shows a much lower overpotential of 203 mV at the current density of 50 mA cm^−2^, which is much better than that of Ir/C. In addition, the Tafel slope of (Ni_0.7_Fe_0.3_)C_2_O_4_ catalyst is about 43 mV dec^−1^, which is lower than that of Ir/C (68 mV dec^−1^). This result demonstrates that Fe cationic doping will enhance charge transfer and mass diffusion.

### Conclusion and Perspectives

Cation‐tuning engineering is an effective strategy to alter the electronic structures of transition metal‐based catalysts for enhanced performance in ORR and OER. Thanks to the capabilities in atomic‐scale regulation and charge‐transfer redistribution, the development of cation vacancies/defects strategy can give us insight into the origin of the activity of electrocatalysts and help us to rationally design more efficient low‐cost electrocatalysts. These cationic‐tuned transition metal‐based catalysts have shown great potential in the ORR, the OER, and to practical applications in Zn‐air batteries. The advantages of such cation vacancies/defects and cation‐doping engineering were discussed and recent advancements of their application for the ORR, the OER, and Zn‐air batteries were reviewed. Although significant efforts have been made, some challenges and opportunities should be paid attention to in the future.

1. The potential of cation‐tuning mechanisms in other electrochemical applications should be explored. It has been proved that in alkaline environment, the distribution and chemical state of cation‐tuning can affect the activity and selectivity of electrocatalysts for oxygen‐related reactions via altering the adsorbed/desorbed behavior of reactants, intermediates, and products. Similar effects can likely be achieved for metal oxide catalysts for OERs in acidic and neutral media, where the intrinsic activities are quite low. It is well‐known that many precious‐metal‐free metal oxides are not thermodynamically stable under acidic media.^[41]^ As the cation doping can tune their coordination environment, and thus it is expected to promote the intrinsic stability of metal oxides in acidic environment. Moreover, the cathode materials of Li/Na ion batteries usually also contain transition metals, such as Co, Fe, Ni, etc.^[42]^ It would be interesting to find out if cationic engineering of these cathode materials can also improve their capacity and stability.
2. Advanced in situ techniques should be developed to monitor the cation vacancies during electrochemical tests, as the cation vacancies/defects and/or cation doping may be unstable. It is well‐known that the electrochemical reactions involve the adsorbed/desorbed intermediates process,^[43]^ and its evolution configuration may result in the disappearance and/or evolution of vacancies and defects. Therefore, in situ monitoring of the change of cation vacancies will be of critical significance for better understanding the reaction mechanisms, especially the in situ measurements by high‐angle annular dark‐field scanning transmission electron microscope (HAADF‐STEM) for cation vacancy evolution processes and X‐ray absorption spectroscopy (XAS) for the coordination environment change of cation vacancies. Besides, other structural characterization techniques, such as Raman, Fourier transform infrared (FTIR) spectroscopy, and electron spin resonance spectroscopy can also be developed for probing the structural evolution of cation vacancies catalysts.
3. Computational calculation has been widely employed on metal oxide catalysts to gain insight into the reaction mechanism. With the development of a deep understanding of metal oxide electrocatalysts, increasing reports have demonstrated that the catalyst itself probably occurs in a dynamic transformation during the catalytic process, especially in the OER. However, the majority of the models for computational analysis are built based on the original catalyst structure. So DFT calculation models established on the real active structure during electrocatalysis are demanded. Additionally, these emerging computer technologies, including machine learning, artificial intelligence, and big data analysis are encouraged to be used in the prediction and simulation of high‐performance metal oxide catalyst applications. These techniques can be considered indispensable in promoting the development of metal oxide electrocatalysts in the future.
4. Though impressive progress has been achieved in metal oxides for oxygen electrocatalysis, most of these studies are still on a laboratory scale. Currently, the synthesis of these cation‐tuning transition metal‐based catalysts usually includes electrochemical reduction, thermal annealing, hydrogenation, wet chemical method, etc. All the related synthetic conditions should be adjusted for mass production. Fussy and hazardous approaches should be avoided, and more efforts should be devoted to the development of facile and environmentally friendly synthetic processes. From this point of view, electrochemical synthesis is regarded as an effective and environment‐friendly way for scaled‐up production, because its parameters, including pH, type of solvents, additives, etc, are feasible to be controlled. Additionally, there is still a gap in electrode performance assessment between fundamental research and practical applications. Parameters and properties such as mass loading of catalysts and long‐term stability in future studies should be comparable to the required amount in practical industrial products.

## Conflict of interest

The authors declare no conflict of interest.

1

## Data Availability

Data sharing is not applicable to this article as no new data were created or analyzed in this study.
